# Short-term variation in abundance of four *Acartia* species (Copepoda, Calanoida) in a eutrophic bay

**DOI:** 10.7717/peerj.10835

**Published:** 2021-03-10

**Authors:** Seo Yeol Choi, Min Ho Seo, Ho Young Soh

**Affiliations:** 1 Department of Ocean Integrated Science, Chonnam National University, Yeosu, Republic of Korea; 2 Marine Ecology Research Center, Yeosu, Republic of Korea

**Keywords:** Zooplankton, Gamak Bay, Interspecific coexistence, Niche separation, Generalized additive models

## Abstract

The short-term variation in the abundance of *Acartia* copepods in the eutrophic Gamak Bay of South Korea was investigated with weekly measurements from October 2007 to September 2008. During this period, four *Acartia* species (*A. erythraea*, *A*. *ohtsukai*, *A*. *omorii*, and *A*.* sinjiensis*) were recorded as showing seasonally different peak abundance. The abundance of *A*. *erythraea* and *A*. *sinjiensis* was high in autumn, whereas that of *A*. *omorii* was high from winter to spring. In summer, *A. erythraea*, *A*. *ohtsukai,* and *A*.* sinjiensis* coexisted at peak abundance significantly related to water temperature and salinity. Results from the response curves of the four *Acartia* species to water temperature and salinity suggest that *A*. *erythraea* and *A*. *sinjiensis* increased in abundance at water temperatures >18 °C, whereas *A*. *ohtsukai* increased in abundance at water temperatures >27 °C. The occurrence of *A*. *erythraea*, *A*. *ohtsukai*, and *A*. *sinjiensis* decreased with increasing salinity, but chlorophyll-*a* concentration showed no effect on occurrence. Despite these findings, the coexistence of the three ecologically similar species may be due to prey abundance in summer and autumn (chlorophyll-*a* concentration >10 µg L^−1^). Notably, the wide range of the response curve of *A. omorii* indicates its occurrence at higher salinity levels than other species.

## Introduction

In marine ecosystems, zooplankton feed on phytoplankton and play an important role as biological connectors, linking primary producers to predators higher in the marine food chain (Hirst, Roff & Lampitt, 2003; Turner, 2004). Therefore, it is important to conduct comprehensive studies on zooplankton to better understand the structure and function of marine ecosystems and estimate the variance in the abundance of higher- and lower-level consumers (Richardson & Schoeman, 2004).

Copepods, a type of zooplankton, are commonly found in oceans worldwide and are generally well-studied. Research on copepod physiology includes respiration and other metabolic processes and behaviours, such as mating and predator avoidance (Gaudy et al., 2003; Meerhoff et al., 2007; Hansen & Drillet, 2013). However, the primary processes that promote the coexistence of ecologically similar species and maintain high marine diversity levels have not received considerable research attention (Chesson, 2000; Helaouët & Beaugrand, 2007). In particular, niche separation is considered a key process that facilitates the coexistence and diversity of ecologically and functionally similar species (Greenwood, 1981; Lakkis, 1994; Lindegren et al., 2020). Notably, niche separation generally reduces competition for resources, such as food and habitat, which inevitably leads to some species dominating and others being eliminated due to their inability to compete, ultimately causing biodiversity loss (Greenwood, 1981; Mayfield & Levine, 2010). Although cross-species competition can be decreased in several ways, modifying the resource requirements of competing species can reduce or minimise overlap between fundamental niches (Hutchinson, 1957).

Previous studies have indicated that *Acartia* species can coexist in semi-enclosed estuarine areas at seasonally high abundance (Greenwood, 1981; Ueda, 1987; Escamilla, Ordóñez López & Suárez-Morales, 2011) due to niche separation for resources such as food and habitat (Gaudy, Cervetto & Pagano, 2000). Niche separation and the continued coexistence of *Acartia* species are believed to result from slight differences between species in terms of adaptability and preference to specific environmental conditions (Lakkis, 1994). Furthermore, physical (water temperature, salinity, and tidal exchange) and biological (feeding, predation, and competition) factors play a pivotal role in determining the spatio-temporal distribution of copepods within the genus *Acartia* (Trinast, 1975; Jonsson & Tiselius, 1990; Buskey, 1994; Lakkis, 1994; Gaudy, Cervetto & Pagano, 2000). To better understand the environmental factors that enable the coexistence of various *Acartia* species in the eutrophic Gamak Bay of South Korea, we investigated the relationship between the short-term variation in abundance of four *Acartia* species and relevant environmental factors.

**Figure 1:**
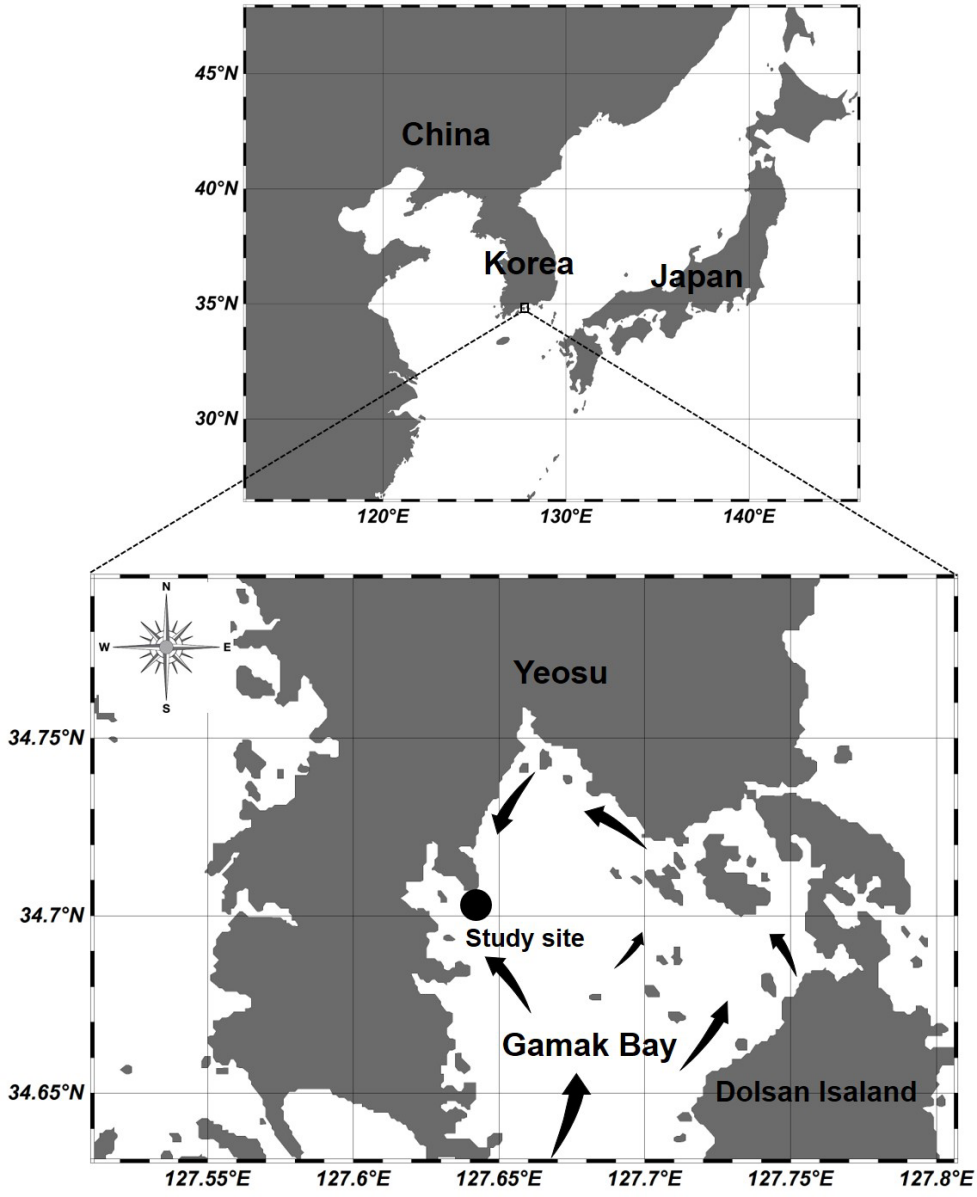
Location of the sampling station at Gamak Bay, including direction of tidal flood current in Gamak Bay (Lee, 1992).

## Materials & Methods

Gamak Bay is an oval and semi-closed bay located in the south of Yeosu Peninsula, South Korea. It has an average depth of 9 m. The direction of the tidal current is shown in Fig. 1, and the water in Gamak Bay is exchanged through the southern entrance of the main bay and the narrow northeast waterway (Lee, 1992; Koo, Je & Shin, 2004). Fishing and shellfish farming are the prevalent commercial activities at Gamak Bay, and this bay has been a traditional fishing site due to its high primary productivity (Lee & Cho, 1990). The centralization of surrounding industrial facilities and farms reduces the water circulation between the surface and bottom waters in the inner bay, i.e., northwestern Gamak Bay (Lee & Cho, 1990; Kim et al., 2006). In particular, hypoxia frequently occurs in the summer when the water temperature is high (Yoon, 2000). To investigate the abundance of *Acartia* species (*A*. *erythraea*, *A*. *ohtsukai*, *A*. *omorii*, and *A*. *sinjiensis*) in northern of Gamak Bay (34°44′44″N, 127°39′28″E), Yeosu, South Korea, weekly samples were collected at high tide from October 2007 to September 2008. Zooplankton samples were towed vertically from the sea bottom to the surface in a fixed station with an average depth of 9 m at a speed of 1 m/s using a conical net (mesh size 200 µm, mouth diameter 45 cm). The collected samples were immediately fixed in a neutralised formaldehyde solution on-site, resulting in a seawater-formalin concentration of approximately 5%. To calculate species composition and abundance, a Folsom-type plankton splitter was used to divide samples by 1/2 to 1/64. A stereomicroscope (Nikon SMZ 1000; Nikon, Japan) in a Bogorov counting chamber was used to identify and count *Acartia* species, and a high-magnification optical microscope (Nikon ECLIPSE 80i; Nikon, Japan) was used for a more detailed identification of *Acartia* species after dissection and mounting of characteristic appendages. The developmental life stages of *Acartia* species were classified as copepodites (copepodids I–V) and adults based on morphological characteristics described by Huys & Boxshall (1991). Sampled individuals were converted into the respective number of individuals per cubic metre (indiv m^−3^) using rotation counts of a flowmeter (Hydro-Bios 438115) attached to the net mouth. A survey of physical (temperature and salinity) and biological (chlorophyll-*a*) factor was conducted on the surface (0.5 m) and bottom layers (B-1 m). Water temperature and salinity were measured using an YSI multimeter (Model 63; Xylem Inc., Yellow Springs, OH, USA). To measure the chl-*a* concentration, 500 mL of surface and bottom water samples were collected using a Niskin sampler and filtered through a 0.45-µm Advantec membrane filter (Advantec MFS Inc., Tokyo, Japan). Filters were stored in cold and dark conditions before further laboratory analyses. Pigments were extracted in 90% acetone, and chlorophyll-*a* concentration was measured spectrophotometrically according to the method described by Lorenzen (1967).

### Statistical analyses

To analyse the effects of environmental factors on the single-cycle variation of four *Acartia* species, we performed a redundancy analysis (RDA) using CANOCO software (version 4.5). Statistical analyses included log_10_ (*x* + 1) transformations to improve normality and homogeneity of variance. Environmental variables were selected manually using forward selection in RDA, and only variables significantly related to taxonomic abundance (*p* < 0.05) were selected using the Monte Carlo permutation test. RDA is a suitable method for revealing correlations between species abundance and the surrounding environment (Lepš & Šmilauer, 2003). Abundance data were used for *Acartia* species during the survey period, and the data on environmental factors (water temperature, salinity, and chl-*a* concentration) are presented as the average of the values measured at the surface and bottom layers.

The generalised additive model (GAM) provides a suitable and commonly used tool for investigating the nonlinear dependence of ecological data. To simplify the GAM, we used a maximum of three degrees of freedom to limit the response of each species to the curve. Furthermore, a GAM regression was performed using the CanoDraw module from the CANOCO software package (Lepš & Šmilauer, 2003).

## Results

### Environmental variation

**Figure 2:**
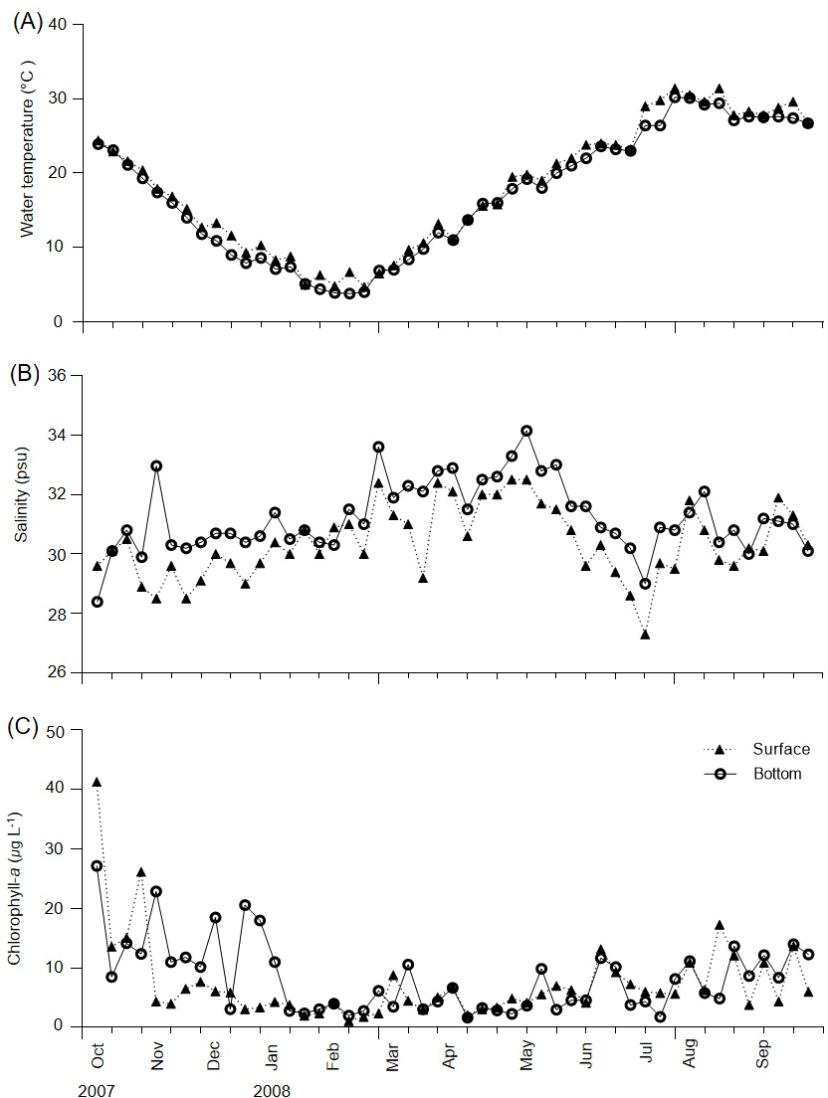
Weekly variations of environmental variables in Gamak Bay. (A) Water temperature (°C), (B) salinity (psu), and (C) chlorophyll-*a* (µg L^−1^).

During the survey period, water temperature varied from 4.7–31.4 °C in the surface layer and 3.8–30.2 °C in the bottom layer (Fig. 2A). The lowest temperature (3.8 °C) was observed in the bottom layer in February, whereas the highest water temperature (31.4 °C) was recorded in the surface layer in July. No major differences in water temperature were recorded between the surface and bottom layers, with the exception of 18 July 2007 (3.4 °C). Salinity varied from 27.3–32.5 psu in the surface layer and 28.4–34.2 psu in the bottom layer (Fig. 2B). Differences in salinity between the surface and bottom layers ranged from 0–4.5 psu (3 November), with a mean difference of 0.9 psu.

Chl-*a* concentrations varied from 1.1–41.3 µg L^−1^ in the surface layer and 1.7–27.2 µg L^−1^ in the bottom layer, with mean concentrations of 7.4 and 8.3 µg L^−1^ in the surface and bottom layers, respectively (Fig. 2C). Furthermore, the surface and bottom layers showed high chl-*a* concentrations from August to December and chl-*a* concentrations of ≥ 10 µg L^−1^ occurred 9 and 14 times in the surface and bottom layers, respectively. Maximum chl-*a* concentrations of 41.3 µg L^−1^ in the surface layer and 27.2 µg L^−1^ in the bottom layer were recorded on 5 October; the maximum chl-*a* concentration in the surface layer reduced before that in the bottom layer. A minimum surface layer chl-*a* concentration of 1.1 µg L^−1^was observed on 13 February, with a minimum bottom layer chl-*a* concentration of 1.7 µg L^−1^ observed on 11 April.

### Variation in the abundance of *Acartia* species

**Figure 3:**
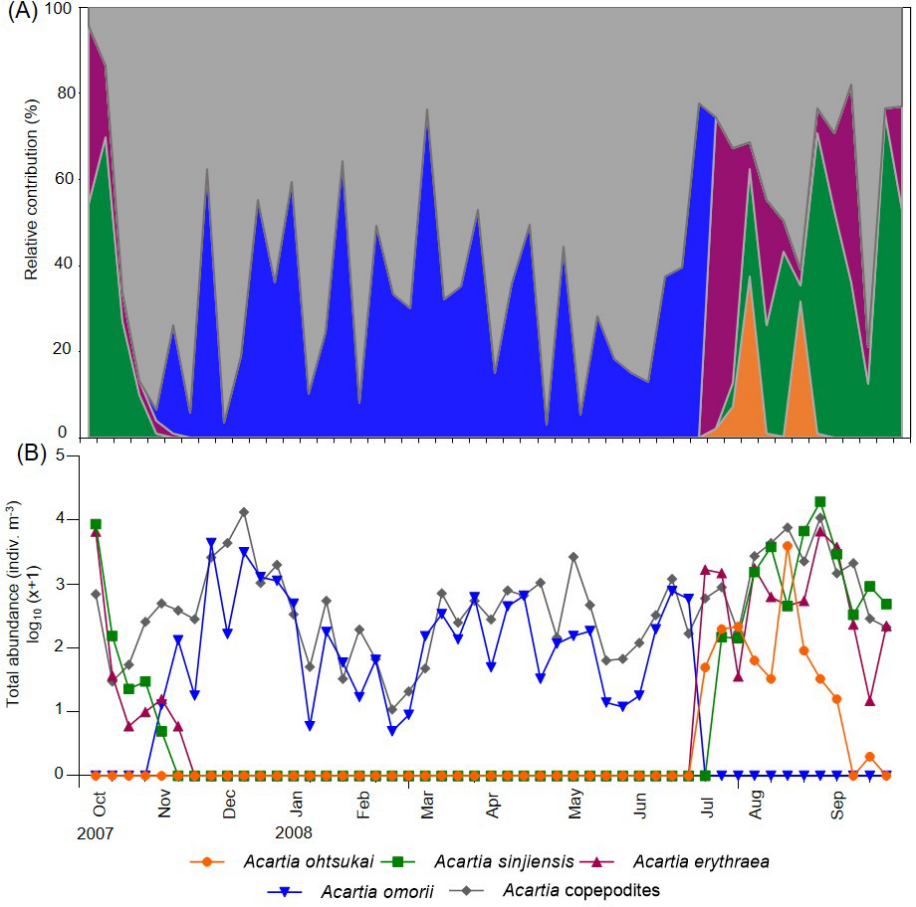
Weekly variations in relative contribution and total abundance of *Acartia* species in Gamak Bay. (A) relative contribution (%) of *Acartia ohtsukai* (orange), *Acartia sinjiensis* (green), *Acartia erythraea* (purple), *Acartia omorii* (blue) and *Acartia* copepodites (grey), (B) total abundance (indiv m^−3^).

Four *Acartia* species (*A*. *erythraea*, *A*. *ohtsukai*, *A*. *omorii*, and *A*. *sinjiensis*) were detected in Gamak Bay during the study period. The abundance of *A*. *erythraea* ranged from 0–6,734 indiv m^−3^ (mean: 497 indiv m^−3^), with the highest abundance peak recorded on 27 August. Starting in June, the abundance remained high, with a second peak recorded in early October (Fig. 3). The abundance of *A*. *ohtsukai* ranged from 0–3,976 indiv m^−3^ (mean: 96 indiv m^−3^), with a maximum abundance recorded on 13 August. The abundance of this species gradually increased starting in July and peaked in August; the species disappeared in mid-September. The abundance of *A*. *omorii* ranged from 0–4,391 indiv m^−3^ (mean: 319 indiv m^−3^), peaking on 25 November. The species gradually increased in abundance from November onwards but disappeared in mid-July. Lastly, the abundance of *A*. *sinjiensis* ranged from 0–19,561 indiv m^−3^ (mean: 940 indiv m^−3^), reaching the highest abundance peak on 27 November. This species had the highest average abundance of the four *Acartia* species. Notably, the overall abundance of *Acartia* copepodites ranged from 11–13,395 indiv m^−3^ (mean: 1,435 indiv m^−3^), with a maximum peak recorded on 11 December. Starting in December, the abundance remained low, with a minimum abundance recorded in February (Fig. 3).

### RDAs and species response curves (water temperature and salinity)

**Figure 4:**
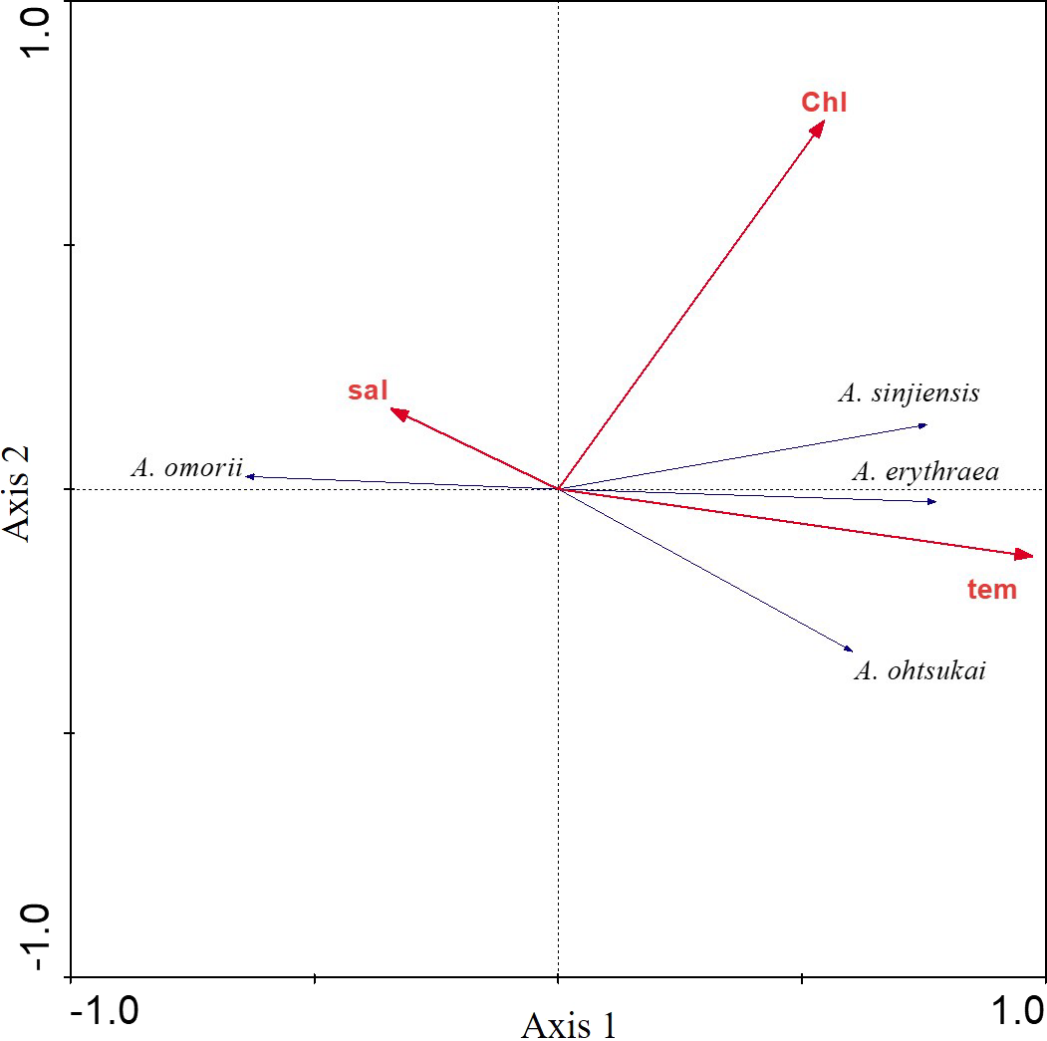
Redundancy analysis (RDA) biplot for environmental factors and *Acartia* species. Labels are: sal: salinity; chl: chlorophyll-*a* concentration; tem: water temperature; *A*. *omorii*: *Acartia omorii*; *A*. *sinjiensis*: *Acartia sinjiensis*; *A*. *erythraea*: *Acartia erythraea*; *A*. *ohtsukai*: *Acartia ohtsukai*.

The relationship between the four recorded *Acartia* species and three environmental factors (water temperature, salinity, and chl-*a* concentration) was investigated using RDAs. The first eigenvalues explained 50.3% of the cumulative variance of species data (Table 1). Furthermore, the species-environment correlations of axes 1 (0.792) and 2 (0.567) were relatively high. The three environmental factors in axes 1 and 2 explained 99.5% of the variance in *Acartia* species. RDAs showed that *A*. *omorii* had a positive correlation with salinity, whereas *A*. *erythraea*, *A*. *ohtsukai*, and *A*. *sinjiensis* were positively correlated with temperature but not significantly correlated with chl-*a* concentration (Fig. 4).

**Table 1:** Summary of redundancy analysis (RDA) for four *Acartia* species and different environmental factors in Gamak Bay.

	**Axes-1**	**Axes-2**	**Axes-3**	**Axes-4**
Eigenvalues	0.485	0.018	0.002	0.294
Species-environment correlations	0.792	0.567	0.303	0.000
Cumulative percentage variance of				
species data	48.5	50.3	50.5	80.0
species-environment relation	95.9	99.5	100	0
Sum of all eigenvalues		1.000		
Sum of all canonical eigenvalues		0.505		

**Figure 5:**
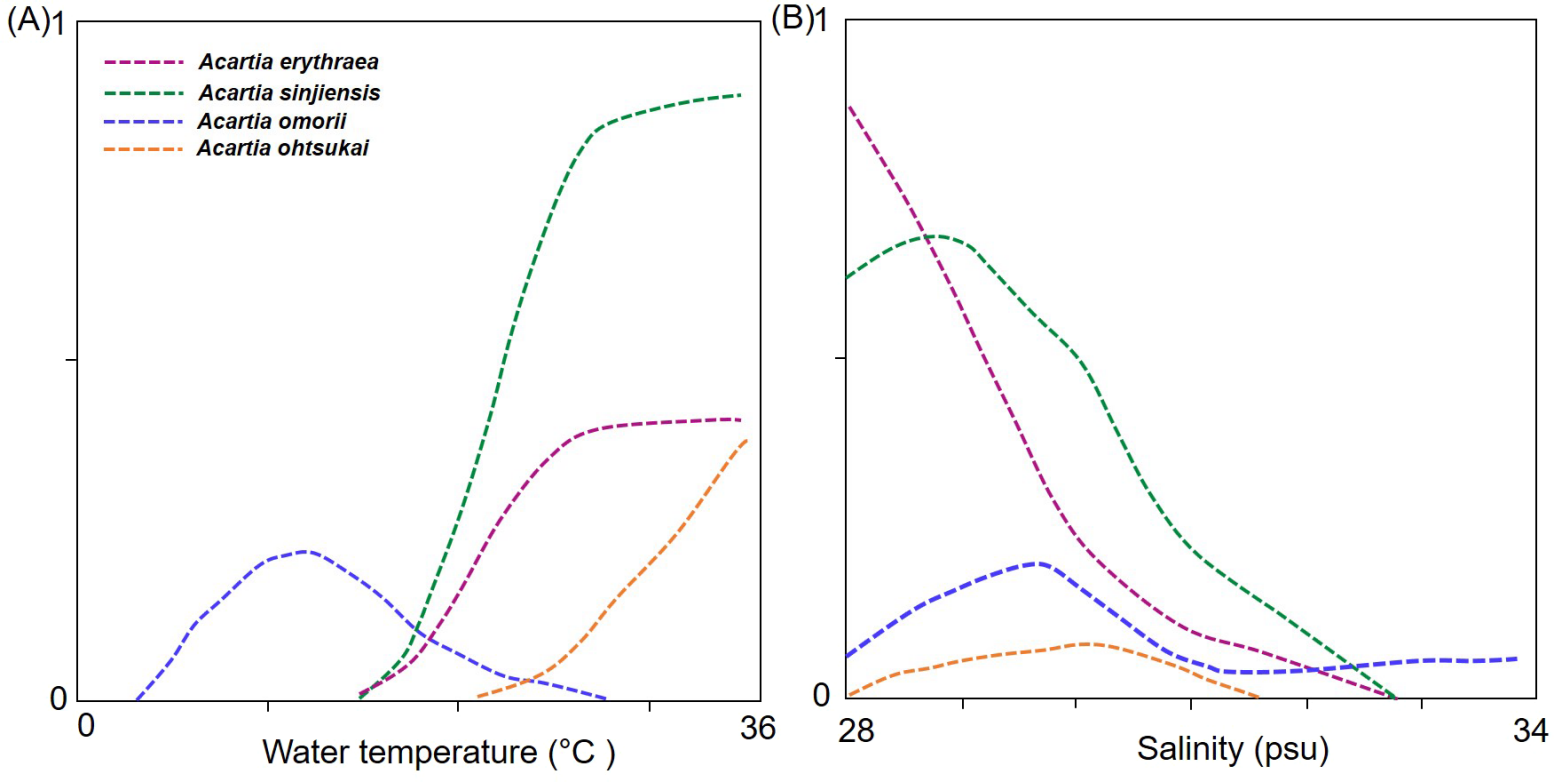
Response curves from generalised additive models (GAM) based on environmental factors. (A) Water temperature (°C), (B) salinity (psu), and abundance of *Acartia* species.

In the response curve models of each species in relation to temperature and salinity, the probability of the occurrence of *A*. *erythraea* and *A*. *sinjiensis* tended to increase at water temperatures >18 °C and salinity levels <31 psu, whereas that of *A*. *ohtsukai* increased at water temperatures >27 °C (Fig. 5). Notably, *A*. *omorii* occurred at lower temperatures and higher salinity concentrations than the other three species.

## Discussion

Four *Acartia* copepods (*A. erythraea*, *A*. *ohtsukai*, *A*. *omorii*, and *A*. *sinjiensis*) were recorded in Gamak Bay, with seasonally different occurrence patterns. *A. erythraea*, *A*. *ohtsukai*, and *A*. *sinjiensis* predominantly coexisted during the high-water temperature season from July to September when salinity levels are low. Of these three species, *A. ohtsukai* disappeared in late September, and *A. erythraea* and *A. sinjiensis* rapidly declined in abundance from the end of October and completely disappeared in December. *A. omorii* replaced the three species from late November to late June (winter to spring) when salinity levels are high. The seasonal succession of *Acartia* species is the outcome of the combined effect of species-specific preferences to varying temperature and salinity regimes in coastal waters (Marques et al., 2006; Park, Suh & Soh, 2015; Zuraire et al., 2018). In the present study, we confirmed the correlation of water temperature and salinity with *Acartia* species (*p* < 0.05). Of the environmental factors investigated, water temperature had the most significant effect on the abundance of *Acartia* species. However, the response curves for water temperature and salinity differed across species (Fig. 5); *A*. *erythraea* and *A*. *sinjiensis* preferred higher temperatures within a wider range (17.7–30.8 °C), and *A*. *ohtsukai* favoured higher temperatures within a narrower range (27.5–30.8  °C). During the study period, *A*. *omorii* preferred low temperatures and appeared in a wide range (4.4–23.8 °C). The derived thermal niches suggest that *A*. *erythraea* and *A*. *sinjiensis* can occur at lower water temperatures than *A*. *ohtsukai* in Gamak Bay during the summer-autumn season.

Results from investigations on the effect of salinity on the abundance of *Acartia* species in estuarine water indicate that salinity affects niche separation between *Acartia* species, particularly in oligohaline to mesohaline regions (Lakkis, 1994; Park, Suh & Soh, 2015). Although the salinity range (27.3–34.2 psu) of the sample site analysed in this study corresponds to a polyhaline region, salinity levels decreased during the summer months after the monsoon, suggesting that temperature may be the most important factor in areas with relatively stable salinity levels. In this context, the wide range of the *A*. *omorii* response curve indicates that the species could be adapted to living in more saline waters than the other species.

Together with temperature and salinity, niche partitioning for food resources is a key process that enables the coexistence and maintenance of biodiversity by reducing interspecific competition (Hutchinson, 1957; Greenwood, 1981; Lakkis, 1994). *Acartia* species that occur together can typically avoid or minimise mutual competition by adopting different spatio-temporal distributions or feeding patterns (Greenwood, 1981; Rollwagen Bollens & Penry, 2003). In Gamak Bay, *Acartia erythraea*, *A*. *ohtsukai*, and *A*. *sinjiensis* occurred together in summer and autumn when chlorophyll-*a* concentrations were high (>11 µg L^−1^), whereas *A*. *omorii* only appeared in winter and spring when chlorophyll-*a* concentrations were <6 µg L^−1^. The body length of *A. ohtsukai* (1.03–1.23 mm) was similar to that of *A. erythraea* (1.1–1.2 mm), whereas *A. sinjiensis* (0.8–1.1 mm) had the shortest body length. In Gamak Bay, high abundance of *Chaetoceros* sp., *Skeletonema costatum, Prorocentrum* spp., *Cochlodinium polykrikoides*, and *Heterosigma akashiwo* (with sizes of 2–50 µm) has been recorded in summer and autumn (Lee et al., 2009). *Acartia* species predate on these diatoms and dinoflagellates. Lee et al. (2012) conducted a study on four copepods (*Acartia hongi*, *A*. *pacifica*, *Calanus sinicus*, and *Paracalanus parvus* s.l.) that feed on similar prey and reportedthat the larger *C*. *sinicus* feed on larger phytoplankton, whereas the other three species prefer phytoplankton of smaller size (<20 µm). Furthermore, Liu et al. (2010) recorded that *A*. *erythraea* prefer for *Prorocentrum minimum*, which belongs to Dinophyceae and is 13–10 µm in size, indicating that prey selectivity across species differs according to prey size. In this context, we suggest that *A*. *erythraea* competes with *A*. *ohtsukai* for similar-sized prey, particularly from August to September. As water temperatures decrease, the competition for prey decreases due to differences in the adaptability of the two species to lower water temperature (Fig. 5). When the *Acartia* copepods are coexist in the same sea area, the strong-tolerant species dominate, and the weaker species are eliminated first (Tranter & Abraham, 1971). *Acartia ohtsukai* was absent during the investigation period (i.e., average water temperature <27 °C). However, niche partitioning could exist for prey resources between *A*. *erythraea* and *A. sinjiensis*, as *Acartia* selectivity for individual prey taxa is the strongest when food is highly abundant and differs according to the body length of the congeners (Rollwagen Bollens & Penry, 2003; Lee et al., 2012).

Although our results indicate a weak relationship between *Acartia* species and chlorophyll-*a* concentration (*p* > 0.05), according to a study by Parsons, Takahashi & Hargrave (2013), obtaining a significant correlation is difficult when simultaneously comparing in-situ food concentrations and populations. This is because of the time lag between food intake and reproduction that reflects population numbers. Greenwood (1981) suggested that all aspects of the biology of a species could be involved in competition, implying that competition for trophic reserves is one of the primary factors of biotic environments. Nutritional niche separation has been suggested as particularly important when information to investigate spatio-temporal differences is insufficient (Mullin, 1966). For example, Christou & Verriopoulos (1993) found that for *A*. *clausi*, the body weight for a given cephalothorax length depends on the amount of available chlorophyll. *Acartia* copepods exist in various sizes, and changes in the quantity or quality of food can affect the prosome to urosome ratio (Gaudy & Verriopoulos, 2004). Therefore, our results indicate that the concentration of prey found in Gamak Bay during the summer months may be sufficient to sustain and enable the coexistence of various *Acartia* species.

In Gamak Bay, we recorded four *Acartia* species as seasonally predominant during the sampling period, although none of the species maintained their populations throughout the entire year. In *Acartia* copepods, the production of dormant eggs is a well-known lifecycle strategy for maintaining population numbers (Grice & Marcus, 1981; Uye, 1985; Castro-Longoria & Williams, 1999). Dormant eggs are produced according to changes in water temperature, and if the appropriate water temperature is reached, these eggs will temporarily hatch and show a repeated generation maintenance strategy (Uye, 1980; Uye, 1985). Considering the general growth pattern of copepods, sudden increases in the population of *Acartia* species can be explained by the temperature-led hatching of dormant eggs. Although the recording of dormant eggs is beyond the scope of our study, the high abundance of *Acartia* species in Gamak Bay indicates that the environmental conditions of the study area support the hatching of dormant eggs.

## Conclusions

Weekly observations revealed a relationship between seasonal changes in *Acartia* species and environmental factors in the eutrophic Gamak Bay. The response curve to water temperature and salinity differed by species, which were divided into summer-autumn (*A*. *erythraea*, *A*. *sinjiensis,* and *A*. *ohtsukai*) and spring-winter (*A*. *omorii*) species. The temperature niches suggested that two species (*A*. *erythraea* and *A*. *sinjiensis*) could occur over a wider range of water temperatures than *A*. *ohtsukai* in Gamak Bay during the summer-autumn season. Notably, the appearance of congeners in the same water area suggests a scenario in which a wide variety of food resources in Gamak Bay can coexist through niche partitioning along with information on food habits.

In conclusion, our results indicate that the coexistence and niche separation of four *Acartia* species are affected by differences in feeding strategies and tolerance to temperature and salinity levels. Niche separation can be explained in terms of different responses to environmental factors. Our study supports the conclusion that changes in key environmental factors are likely to cause niche separation that enables the coexistence of various *Acartia* populations.

##  Supplemental Information

10.7717/peerj.10835/supp-1 Supplemental Information 1 Raw data of Gamak Bay, 2007-2008.

## Additional Information and Declarations

### Data Availability

The following information was supplied regarding data availability: Raw data is available as a Supplemental File.
